# Corticosteroids influence the mortality and morbidity of acute critical illness

**DOI:** 10.1186/cc4971

**Published:** 2006-07-17

**Authors:** Mohamed Y Rady, Daniel J Johnson, Bhavesh Patel, Joel Larson, Richard Helmers

**Affiliations:** 1 Department of Critical Care Medicine, Mayo Clinic College of Medicine, Mayo Clinic Hospital, Mayo Clinic, Phoenix, Arizona, USA; 2 Department of Surgery, Mayo Clinic College of Medicine, Mayo Clinic Hospital, Mayo Clinic, Phoenix, Arizona, USA

## Abstract

**Introduction:**

Use of corticosteroids for adrenal supplementation and attenuation of the inflammatory and immune response is widespread in acute critical illness. The study hypothesis was that exposure to corticosteroids influences the mortality and morbidity in acute critical illness.

**Methods:**

This case–control retrospective study was performed in a single multidisciplinary intensive care unit at a tertiary care institution and consisted of 10,285 critically ill patients admitted between 1 January 1999 and 31 December 2004. Demographics, comorbidities, acute illness characteristics including severity measured by Sequential Organ Failure Assessment, concurrent medications, therapeutic interventions and incidence of infections were obtained from electronic medical records, were examined with multiple regression analysis and were adjusted for propensity of corticosteroid exposure. The primary outcome was hospital death, and the secondary outcome was transfer to a care facility at hospital discharge.

**Results:**

Corticosteroid exposure in 2,632 (26%) patients was characterized by younger age, more females, higher Charlson comorbidity and maximal daily Sequential Organ Failure Assessment scores compared with control patients. Corticosteroids potentiated metabolic and neuromuscular sequels of critical illness with increased requirements for diuretics, insulin, protracted weaning from mechanical ventilation, need for tracheostomy and discharge to a care facility. Early exposure to corticosteroids predisposed to recurrent and late onset of polymicrobial and fungal hospital-acquired infections. Corticosteroids increased the risk for death or disability after adjustments for comorbidities and acute illness characteristics.

**Conclusion:**

Corticosteroids increased the risk for death or disability in critical illness. Hospital-acquired infections and metabolic and neuromuscular sequels of critical illness were exacerbated by corticosteroids. Careful appraisal of the indications for use of corticosteroids is necessary to balance the benefits and risks from exposure in acute critical illness.

## Introduction

Administration of corticosteroids in a variety of settings in acute critical illness has become widespread. Corticosteroids are used therapeutically for relative adrenal insufficiency as well as for the attenuation of the inflammatory and immune response in the critically ill [1]. Early use of corticosteroids has been recommended in sepsis, acute lung injury, acute respiratory distress syndrome and refractory vasodilatory shock [2-5]. The Corticosteroid Randomization after Significant Head Injury study, a large, international, randomized placebo-controlled trial, was terminated after enrolment of 10,000 patients because of an unexpected rise in the death rate after early administration of corticosteroids [6]. That study report raised concerns with regard to the safety of corticosteroids since, up to that time, they had been liberally administered in a variety of life-threatening illnesses with the intent to improve survival. These concerns were substantiated when we observed, in a previous study, that administration of corticosteroids increased the mortality in vasopressor-dependent critical illness [7]. A similar observation of an unexpected increase in mortality from corticosteroids use was also reported from a randomized controlled trial of corticosteroids in late acute respiratory distress syndrome [8].

The morbidity related to metabolic, immune and musculoskeletal side-effects of corticosteroids in noncritical illness has been recognized and has created great interest in developing alternative treatments to avoid these complications. In transplantation practice, the therapeutic use of corticosteroids for immunosuppression has decreased because of the introduction of other therapies targeted against specific cytokines including tumour necrosis factor and interleukins or selective lymphocytes calcineurin inhibition [9,10]. New immunosuppression regimes produced superior allograft survival and yet had fewer side effects than traditional high-dose corticosteroids [11,12]. For autoimmune inflammatory disorders and rheumatologic diseases, the use of corticosteroids has also declined because of better treatment options targeting inflammatory cytokines known to influence the progression of these conditions [13-16].

The use of corticosteroids in noncritical illness has gradually diminished, yet their use in acute critical illness appears to be expanding in relative adrenal insufficiency, sepsis and systemic inflammatory organ injury. This study was designed to address the following questions: What are the frequency and patient characteristics associated with corticosteroid use in acute critical illness? Does the exposure to corticosteroids influence death or disability? What were the mechanisms for the observed effects of corticosteroids in acute critical illness? This study was a retrospective case–control analysis of all admissions to an adult intensive care unit (ICU) with exposure to corticosteroids defining the case group.

## Patients and methods

### Study population

The study was granted approval and exemption by the Mayo Foundation Institutional Review Board. The study was performed at Mayo Clinic Hospital, a 220-bed hospital. Patients (≥ 18 years old) were admitted to a closed, 20-bed, multidisciplinary ICU (medical, surgical and coronary care) between January 1999 and December 2004.

### Data collection

The patient demographics, comorbidities, type of admission, therapeutic interventions, acute diagnosis and disposition at hospital discharge were obtained from electronic medical records, which were interfaced into an institutional replicated database and extracted electronically [17]. The initial admission was designated as the index admission for those patients with multiple hospital admissions during the six years. Comorbidities were determined by Romano and colleagues' criteria to calculate the Charlson comorbidity score [18]. Diagnoses recorded for the index admission were used to develop the acute hospital diagnosis categories [17]. The severity of illness in the ICU was determined by the Sequential Organ Failure Assessment score, calculated based on the graded severity of dysfunction of six organ systems: neurological, pulmonary, cardiovascular, hepatic, renal and coagulation [19,20]. The length of stay and time intervals were calculated in hours and then expressed as fractions of days. Hospital discharge to acute, subacute or long-term nursing care, to inpatient rehabilitation, to long-term ventilation or to other types of extended care facilities was utilized as a surrogate marker for disability.

### Medication

All medication given during the hospital stay was tracked by the pharmacy database in LastWord 4.1 for Windows (IDX Systems Corp., Burlington, VT, USA), which recorded the date and time of administration, the dose administered and the route of administration. Catecholamine infusion included norepinephrine, phenylephrine, epinephrine, dopamine, dobutamine or milrinone. Exposure to corticosteroids was defined as either via intravenous or enteral administration routes. The total cumulative doses of corticosteroids were calculated during the entire hospital stay and are expressed as hydrocortisone equivalents according to the glucocorticoid effect.

### Microbiology and diagnosis of infections

Microbiological cultures were obtained to confirm infection based on established clinical criteria. First, the development of vasodilatory shock: persistent hypotension (mean arterial pressure ≤ 60 mmHg) after adequate volume resuscitation to a central venous pressure between 12 and 15 mmHg [21] and requiring initiation of vasopressor therapy for greater than 24 hours. The second criterion was the presence of two or more of the following criteria for systemic inflammatory response syndrome: temperature >38.5°C or <35.6°C, white cell count >11,000 cells/mm^3 ^or <4,000 cells/mm^3^, immature neutrophils (bands) >10%, respiratory rate >20/minute, PaCO_2 _<35 mmHg or requirement for mechanical ventilation, and heart rate >90/minute [22]. Third was the presence of unexplained acute organ dysfunction not due to underlying disease or medications: confusion, restlessness, altered mental status (acute change from baseline) or oliguria. The final criterion was initiation of empiric antibiotics therapy.

The dates and times of cultures performed, including the specimen source, microscopic examination and the semiquantitative organism count, were stored in an electronic database in LastWord 4.1 for Windows (IDX Systems Corp.). Infection was confirmed by the following: the presence of polymorph nuclear cells in normally sterile body fluid; a culture or Gram stain of blood, sputum, urine or normally sterile body fluid positive for pathogenic microorganisms; and a focus of infection identified by visual inspection (such as, bowel perforation with content at time of surgery, wound with purulent discharge, pulmonary consolidation on thoracic imaging).

Microorganism growth quantified as 2+ or >15 colony-forming units were considered clinically significant. The date and time of hospital admission was considered the reference time point for each patient to determine the temporal characteristics of infection episodes documented during the hospital stay. Hospital-acquired infection was defined as a new episode of infection developing >48 hours from hospital admission. Recurrent infection was indicated by two or more episodes of infections with different microorganisms at separate times during a hospital stay. Polymicrobial infection was defined as the presence of two or more types of microorganisms from a single location of infection at the same time.

### Statistical analysis

Corticosteroid exposure during the hospital stay defined the case group versus the control group. Analysis was performed with Student's *t *test or Wilcoxon's rank sum test when appropriate. Categorical variables were analysed by chi-square test or Fisher's exact test as appropriate. A nonparametric test of the median (number of points above the median) was performed where appropriate for comparison of the length of stay. For group comparison, the exact *P *values were reported for each comparison, and Bonferroni correction was applied because of multiple comparisons so that *P *< 0.001 was considered statistically significant between groups.

Preadmission comorbidities, age, sex, type of admission, use of catecholamines, and respiratory and neurological diagnosis were included in a logistic model to calculate the propensity score (the probability for exposure to corticosteroids) for each study patient. The propensity score was included as a covariate in multiple regression analysis for the primary outcome endpoint, death while in the hospital (hospital death), and for the secondary outcome endpoint, discharge to a care facility (disability). The likelihood ratio test determined the cut-off value for the maximal daily Sequential Organ Failure Assessment score.

Stepwise multiple logistic regressions were performed on three groups of predictors: preadmission comorbidities, type of admission with hospital and intensive care, and hospital diagnosis category. All factors that were significant at *P *< 0.1 from each group were entered into the final logistic models. Calibration of the final logistic models was examined by Hosmer-Lemeshow goodness-of-fit. Discrimination of the logistic models was examined by the area under the receiver operating characteristic curve.

All statistical tests were two-tailed and significance was accepted at *P *< 0.05. Statistical analysis was performed using JMP Statistical software (version 5.1; SAS Institute Inc., Cary, NC, USA).

## Results

### Cohort description

There were 10,258 patients admitted to the ICU, 2,632 (26%) of whom received corticosteroids. Patients receiving corticosteroids were more likely to be younger and female (Table 1). Liver disease, malignancy, chronic obstructive or restrictive pulmonary disease, connective tissue disease and cachexia were more frequent comorbidities in patients who received corticosteroids and were reflected by a higher Charlson Comorbidity score than in control patients. Relative adrenal insufficiency or anti-inflammatory action constituted the majority of indications for corticosteroid use (Table 1). The frequency of medical admissions in the corticosteroid exposure group was higher than the control group (Table 1).

### Corticosteroid exposure and hospital care and intensive care

The maximal daily Sequential Organ Failure Assessment score and also the requirement for mechanical ventilation and mechanical ventilation ≥ 96 hours were higher in patients with corticosteroid exposure. The need for tracheotomy was also higher in these patients with a longer stay in the ICU than in control patients. The first dose of corticosteroids was administered early, and in 90% of patients within 4 days from hospital admission (Table 2). The median exposure time to corticosteroids was 3 days at a median total dose of 900 mg hydrocortisone equivalent (Table 2). Catecholamine infusions, antibacterial, antifungal, diuretics, insulin and proton pump inhibitors or histamine-2 blockers were frequent concurrent medications in patients who received corticosteroids compared with control individuals (Table 2).

Respiratory, neurological, fluid and electrolyte abnormalities, postoperative complications and acute renal failure were more frequent categories of hospital diagnosis in patients exposed to corticosteroids than in the control group. These patients had a longer hospital stay and a higher frequency of death or discharge to a care facility than the control group (Table 2). Hospital death rates were similar for the three groups of indications for corticosteroids: relative adrenal insufficiency, 109 patients (11%); anti-inflammatory effects, 126 patients (10%); and immunosuppression, 26 patients (8%) (*P *= 0.2).

### Corticosteroids and infectious complications and clinical outcome

Corticosteroid exposure in patients was associated with more frequent microbiological cultures with a positive yield than in the control group (Table 3). Corticosteroids were associated with higher frequencies of infection at pulmonary, vascular catheter and central nervous system locations than in the control group (Table 3). Hospital-acquired infections (infection episode >2 days after admission) and recurrent infections (two or more infection episodes during a hospital stay) were more frequent after corticosteroid exposure. Late onset of infection (day of last infection episode during a hospital stay) was also observed in that group (Table 3). Fungal and polymicrobial microorganisms were more likely to be present in patients exposed to corticosteroids (Table 3).

Exposure to any type of corticosteroids increased the adjusted risk for hospital death (Table 4). Exposure to corticosteroids with a total hydrocortisone equivalent >900 mg increased the adjusted risk for hospital discharge to a care facility (Table 4).

## Discussion

The study salient findings were as follows. First, exposure to corticosteroids occurred in 26% of intensive care patients. Many of these patients were already suffering from long-standing comorbidities. A third finding was that corticosteroids increased the risk of death or disability at hospital discharge after adjustment for comorbidities and acute illness characteristics. Finally, early corticosteroid administration exacerbated infectious, metabolic and neuromuscular complications in acute critical illness.

### Infectious complications and mortality after corticosteroids

The study observed that corticosteroids increased the risk for hospital death after considering the confounding effects of comorbidities, the diverse nature and severity of the acute illness and therapeutic interventions in the critically ill. The Corticosteroid Randomization after Significant Head Injury study reported similar findings that early exposure to corticosteroids increased mortality two weeks later [6]. The reason for the observed increase in mortality could not be determined in that study; however, in the present study we identified infectious, metabolic and neuromuscular consequences seen in critical illness that were potentiated by corticosteroids and could explain the higher mortality.

While the anti-inflammatory actions could explain the desirable effects of corticosteroids in acute respiratory distress syndrome, septic shock and autoimmune-mediated organ injury, the same actions could also hinder normal host defence mechanisms to overcome and recover from acute infections. We observed that infectious complications related to corticosteroids had distinctive temporal patterns, locations and microbiology characteristics.

The study found that early exposure to corticosteroids predisposed to late development of hospital-acquired infections, with host-borne microorganisms often seen at sites of invasive instrumentation in critical illness such as pulmonary and vascular access sites. Invasive instrumentation at these sites was necessary because of prolonged intensive care and hospital stays in these patients, which increased their vulnerability to recurrent infections. Corticosteroid exposure encouraged polymicrobial and fungal infections. Our observation concurred with previous findings that the use of corticosteroids increased the incidence of fungal superinfections and contributed to higher mortality in the critically ill [23]. In the present study, the late development of infections during hospital stay also explained the high mortality attributed to corticosteroids. Late onset of sepsis from hospital-acquired infection has been associated with a much higher mortality than sepsis related to infection seen early after hospital admission [24].

Our data suggest that corticosteroids increased the incidence of pulmonary infections. The concurrent use of gastric acid suppression with histamine receptor blockade or proton pump inhibitors, prolonged invasive mechanical ventilation and the use of tracheostomy could have predisposed the study patients to frequent pulmonary infections. Patients treated with corticosteroids were also sicker and therefore required gastric acid suppression more commonly and for a longer time than those not treated.

An interesting finding of the study was the trend for administration of corticosteroids, in particular dexamethasone, to patients with central nervous system infections. Early administration of dexamethasone in adults with acute bacterial meningitis had been recommended in order to suppress leptomeningeal inflammations with the aim of decreasing the mortality and neurological sequelae triggered by this type of infection [25]. We did not observe either a mortality reduction or a better functional recovery linked to corticosteroids use in our study. To the contrary, our data suggested that exposure to corticosteroids was an independent factor to increase the risk for death or disability in the presence of a neurological diagnosis (Table 4).

### Metabolic and neuromuscular sequels of corticosteroids

Corticosteroids exacerbated several metabolic abnormalities induced by acute critical illness. The mineralocorticoid activity of the administered corticosteroids aggravated fluid retention and interstitial oedema in the critically ill. We observed that diuretics were concurrently administered in 65% of patients already on corticosteroids. The simultaneous use of corticosteroids and diuretics to overcome fluid retention was responsible for frequent electrolyte abnormalities in these patients.

Our study observed that the frequency of insulin use for glycaemic control increased with corticosteroid exposure. Administration of insulin with poor glycaemic control could explain the higher mortality related to corticosteroids in our patients. In a previous study, we reported that the use of corticosteroids influenced glucose metabolism, resulting in poor glycaemic control and insulin resistance in the critically ill [26]. The control of blood glucose in acute critical illness has been recommended to improve clinical outcome [27]. Excessive insulin use with poor glycaemic control has been linked to an increased mortality in both medical and surgical critical illness [26,28].

A variety of neuromyopathy disorders have been attributed to corticosteroid use in critical illness that delayed the functional recovery of survivors [29,30]. The present study observed that corticosteroids delayed weaning from mechanical ventilation and increased the need for tracheostomy. The incidence of care dependency and disability requiring discharge to a care facility was also high in these patients. Global and flaccid muscle weakness affecting the limb musculature, truncal musculature and respiratory musculature related to necrotizing myopathy have been reported with corticosteroid use in critical illness [31-34]. Delayed recovery of respiratory neuromuscular function and extremity neuromuscular function has been documented because peripheral nerve abnormalities and skeletal muscle abnormalities persisted for years, raising concerns for irreversible neuromuscular dysfunction related to corticosteroid use in critical illness [34,35].

Corticosteroids increased the frequency of postoperative complications in surgical critical illness. Poor or delayed tissue healing, metabolic abnormalities and hospital-acquired infections could explain the vulnerability to complications after surgical procedures in these patients.

### Study limitations

This study was retrospective and performed at a single tertiary care ICU. The patient case mix, the spectrum of comorbidities and hospital care had confounding effects on both the mortality and morbidity observed in this study, and therefore could limit extrapolation of the findings to other practice settings. The study could not define clinical benefit or indications for use of corticosteroids in these patients because of the non-intervention design. The total cumulative dose of corticosteroids given during the hospital stay did not take into account exposure to corticosteroids before hospital admission, which could influence the relationship with clinical outcome. The multivariable analysis had major limitations since unmeasured significant confounding factors might not have been taken into account in this study. Transfer to a care facility was utilized as a measure of delayed recovery and significant disability at hospital discharge. That type of evaluation could have missed the disabilities suffered by survivors who were discharged home, and therefore the reported magnitude of the effect of corticosteroids on disability would have been underestimated.

## Conclusion

Corticosteroids increase the risk for death or disability in acute critical illness. Early exposure to corticosteroids increases the frequency of late hospital acquired infections, polymicrobial infections and fungal infections during the hospital stay. Corticosteroids exacerbate metabolic and neuromuscular sequels of critical illness. Careful appraisal of the indications for use of corticosteroids is necessary to balance the benefits and risks from exposure in acute critical illness.

## Key messages

• 	Corticosteroids increased the risk for death or disability in critical illness.

• 	Corticosteroids exacerbated hospital-acquired infections, and metabolic and neuromuscular sequels of critical illness.

• 	Careful appraisal of the indications for corticosteroids use in critical illness is necessary to balance the benefits and risks from exposure.

## Abbreviations

ICU = intensive care unit.

## Competing interests

The authors declare that they have no competing interests.

## Authors' contributions

The authors attest they have made substantial contributions to conception and design, or acquisition of data, or analysis and interpretation of data; that they have been involved in drafting the manuscript or revising it critically for important intellectual content; that they have given final approval of the version to be published; and that they have participated sufficiently in the work to take public responsibility for appropriate portions of the content. MYR had full access to all of the data in the study and takes responsibility for the integrity of the data and the accuracy of the data analysis. All authors read and approved the final manuscript

## Figures and Tables

**Table 1:** Preadmission comorbidities and types admission of the study cohort

	Control group (*n *= 7,626)	Corticosteroid exposure group (*n *= 2,632)	*P*
Age (years)	72 (48–84)	67 (43–82)	0.0001
Female	2,860 (38)	1,147 (44)	0.0001
Body mass index (kg/m^2^)	26 (20–35)	26 (20–35)	0.8
Peripheral arterial disease	777 (10)	221 (8)	0.008
Cerebral vascular disease	159 (2)	23 (1)	0.0001
Neurological disease (degenerative brain disease)	1,048 (14)	396 (15)	0.1
Congestive heart failure	974 (13)	361 (14)	0.2
Hypertension	3,687 (48)	995 (38)	0.0001
Diabetes mellitus	1,359 (18)	436 (17)	0.1
Liver disease	636 (8)	351 (13)	0.0001
Malignancy	1,179 (15)	685 (26)	0.0001
Chronic obstructive pulmonary disease	870 (11)	565 (21)	0.0001
Pulmonary disease (restrictive and obstructive)	1,025 (13)	663 (25)	0.0001
Renal disease	577 (8)	223 (8)	0.9
Connective tissue disease	88 (1)	197 (7)	0.0001
Cachexia	157 (2)	126 (5)	0.0001
Charlson comorbidity score	1 (0–4)	2 (0–5)	0.0001
Indications for corticosteroids			
Relative adrenal insufficiency	-	1,022 (39)	-
Anti-inflammatory action	-	1,270 (48)	-
Immunosuppression	-	340 (13)	-
Types of admission			
Coronary care unit	1,561 (20)	211 (8)	0.0001
Medical	2,623 (34)	1,168 (44)	0.0001
Surgical	3,442 (46)	1,253 (48)	0.1

Data presented as median (10–90% percentiles) or actual numbers (percentage).

**Table 2:** Acute illness characteristics including hospital care, intensive care and outcome for the study cohort

	Control group (*n *= 7,626)	Corticosteroid exposure group (*n *= 2,632)	*P*
Hospital and intensive care			
Maximal daily Sequential Organ Failure Assessment	3 (0–9)	4 (1–11)	0.0001
Mechanical ventilation	1,889 (25)	765 (29)	0.0001
Mechanical ventilation ≥ 96 hours	297 (4)	372 (14)	0.0001
Tracheostomy	124 (2)	204 (8)	0.0001
Intensive care stay (days)	1.2 (0.2–4.6)	1.9 (0.3–9.6)	0.0001
Hospital stay (days)	5.1 (1.3–12.4)	6.8 (2.0–19.0)	0.0001
Corticosteroid exposure			
Hydrocortisone	0	293 (11)	
Dexamethasone	0	783 (30)	
Methyl prednisone or prednisone	0	1,556 (59)	
Start day of corticosteroids	0	0 (0–4)	
Corticosteroid exposure time (days)	0	3 (1–13)	
Total hydrocortisone equivalent (mg)	0	900 (100–6,600)	
Concurrent medication			
Catecholamine infusions	2,973 (39)	1,291 (49)	0.0001
Antibacterial antibiotics	5,217 (68)	2,318 (88)	0.0001
Antifungal antibiotics	419 (5)	557 (21)	0.0001
Diuretics	4,360 (57)	1,702 (65)	0.0001
Insulin	2,424 (32)	1,266 (48)	0.0001
Proton pump inhibitors or Histamin-2 blockade	5,142 (67)	2,205 (84)	0.0001
Hospital diagnosis category			
Cardiovascular	2,348 (31)	790 (30)	0.5
Respiratory	1,346 (18)	774 (29)	0.0001
Neurological	876 (11)	440 (17)	0.0001
Fluid and electrolytes abnormalities	1,504 (20)	694 (26)	0.0001
Postoperative complications	856 (11)	406 (15)	0.0001
Gastrointestinal	1,023 (13)	381 (14)	0.2
Acute renal failure	375 (5)	265 (10)	0.0001
Hospital outcome			
Hospital death	327 (4)	261 (10)	0.0001
Discharge to a care facility	1,562 (20)	762 (29)	0.0001

Data presented as median (10–90% percentiles) or actual numbers (percentage).

**Table 3:** Microbiological characteristics of the study cohort

	Control group (*n *= 7,626)	Corticosteroid exposure group (*n *= 2,632)	*P*
Microbiology			
Number of patients with cultures	3,185 (42)	1,764 (67)	0.0001
Infection confirmed	1,785 (23)	1,215 (46)	0.0001
Location of infection	*n *= 1,785	*n *= 1,215	
Pulmonary	578 (32)	489 (40)	0.0001
Abdomen	197 (11)	147 (12)	0.4
Genital–urinary tract	703 (39)	425 (35)	0.02
Bloodstream	500 (28)	376 (31)	0.09
Soft tissue	165 (9)	90 (7)	0.08
Vascular catheters	156 (9)	158 (13)	0.0002
Skeletal	54 (3)	24 (2)	0.08
Central nervous system	46 (3)	47 (4)	0.05
Head and neck	17 (1)	21 (2)	0.07
Other	16 (1)	9 (1)	0.7
Temporal characteristics	*n *= 1,785	*n *= 1,215	
Infection episode ≤ 2 days after admission	1,228 (69)	820 (67)	0.5
Infection episode >2 days after admission	883 (49)	669 (55)	0.003
Recurrent infection^a^	633 (35)	521 (43)	0.0001
Day of last infection episode during a hospital stay	2 (0–13)	3 (0–19)	0.003
Interval between first and last infection episodes (days)	0 (0–10)	0 (0–15)	0.0001
Interval between first and last infection episodes >2 days	434 (24)	402 (33)	0.0001
Types of microorganisms (culture or microscopy)	*n *= 1,785	*n *= 1,215	
Bacterial			
Gram-positive cocci	1,255 (70)	903 (74)	0.02
Gram-negative bacilli	744 (42)	487 (40)	0.4
Other types of bacilli^b^	299 (17)	221 (18)	0.3
Anaerobes^c^	182 (10)	112 (9)	0.4
Gram-negative cocci	66 (4)	38 (3)	0.4
Fungal	404(23)	508 (42)	0.0001
Number of types of microorganisms per location	1 (1–4)	2 (1–6)	0.0001
Polymicrobial infection^d^	838 (47)	683 (56)	0.0001

Data presented as median (10–90% percentiles) or actual numbers (percentage). Cultures included blood, sputum, urine, normally sterile body fluid or tissue. ^a^Two or more infection episodes during a hospital stay. ^b^*listeria*, *Corynebacteria*, *Actinomyces*, *bacilli Sp*., *Legionella *and *Mycobacterial *species; ^c^*Clostridia, Bacteroids*, *Peptostreptococci*, *and Peptococci species*.. ^d^Two or more types of organisms present at the same location site of infection.

**Table 4:** Multiple regression analysis of factors predicting hospital death and discharge to a care facility

	Wald chi-square	Odds ratio (95% confidence interval)	*P*
**Hospital death**^**a**^			
Preadmission comorbidities			
Age per decade	22	3.2 (1.9–5.6)	<0.0001
Female	6	1.3 (1.0–1.5)	0.0176
Malignancy	18	1.7 (1.3–2.2)	<0.0001
Hospital and intensive care			
Medical admission	112	3.2 (2.6–3.9)	<0.0001
Mechanical ventilation	74	2.6 (2.1–3.2)	<0.0001
Maximal daily Sequential Organ Failure Assessment score >8	136	3.8 (3.0–4.8)	<0.0001
Corticosteroid exposure (hydrocortisone, dexamethasone, methyl prednisone or prednisone)	11	1.4 (1.1–1.7)	0.001
Hospital diagnosis category			
Acute renal failure	81	3.0 (2.4–3.9)	<0.0001
Cardiovascular	62	2.2 (1.8–2.7)	<0.0001
Neurological	69	2.6 (2.1–3.3)	<0.0001
Respiratory	16	1.5 (1.2–1.8)	<0.0001
**Discharge to care facility**^**b**^			
Preadmission comorbidities			
Age per decade	137	7.9 (5.6–11.1)	<0.0001
Female	19	1.3 (1.1–1.4)	<0.0001
Cerebral vascular disease	24	1.6 (1.3–2.0)	<0.0001
Degenerative brain disease	212	2.7 (2.3–3.0)	<0.0001
Congestive heart failure	13	1.3 (1.1–1.5)	0.00040
Chronic obstructive pulmonary disease	4	1.1 (0.9–1.3)	0.05880
Cachexia	11	1.6 (1.2–2.1)	0.00040
Diabetes mellitus	6	1.2 (1.0–1.4)	0.01
Hospital and intensive care			
Medical admission	118	1.9 (1.7–2.1)	<0.0001
Corticosteroid exposure			
Total hydrocortisone equivalent >900 mg	6	1.2 (1.0–1.4)	0.01
Maximal daily Sequential Organ Failure Assessment score ≥ 4	21	1.3 (1.2–1.5)	<0.0001
Hospital-acquired infection	124	2.2 (1.9–2.6)	<0.0001
Antifungal antibiotics	10	1.3 (1.1–1.6)	0.00160
Tracheostomy	63	2.9 (2.3–3.8)	<0.0001
Hospital diagnosis category			
Neurological	57	1.7 (1.5–2.0)	<0.0001
Respiratory	10	1.2 (1.1–1.4)	0.00200
Postoperative complications	28	1.5 (1.3–1.7)	<0.0001
Fluid and electrolyte abnormalities	13	1.2 (1.1–1.4)	0.00030

^a^The calibration of the multiple logistic model (lack of fit *P *= 0.9) and discrimination characteristic (area under the receiver operating characteristic curve = 0.88); *P *< 0.01 for all predictive factors. ^b^The calibration of the multiple logistic model (lack of fit *P *= 0.9) and discrimination characteristic (area under the receiver operating characteristic curve = 0.76); *P *< 0.01 for all predictive factors.
